# Macroscopic and Histopathologic Findings From a Mass Stranding of Rough-Toothed Dolphins (*Steno bredanensis*) in 2005 on Marathon Key, Florida, USA

**DOI:** 10.3389/fvets.2020.00572

**Published:** 2020-09-02

**Authors:** Ruth Y. Ewing, David S. Rotstein, William A. McLellan, Alexander M. Costidis, Gretchen Lovewell, Adam M. Schaefer, Carlos H. Romero, Gregory D. Bossart

**Affiliations:** ^1^Southeast Fisheries Science Center, National Marine Fisheries Service, National Oceanographic and Atmospheric Administration, Miami, FL, United States; ^2^Marine Mammal Pathology Services, Olney, MD, United States; ^3^Department of Biology and Marine Biology, University of North Carolina Wilmington, Wilmington, NC, United States; ^4^Virginia Aquarium & Marine Science Center, Virginia Beach, VA, United States; ^5^Directorate of Marine Biology and Conservation, Mote Marine Laboratory, Sarasota, FL, United States; ^6^Center for Coastal Research-Marine Mammal Research and Conservation Program, Harbor Branch Oceanographic Institute, Florida Atlantic University, Fort Pierce, FL, United States; ^7^Department of Infectious Diseases and Pathology, College of Veterinary Medicine, University of Florida, Gainesville, FL, United States; ^8^Georgia Aquarium, Atlanta, GA, United States; ^9^Division of Comparative Pathology, Department of Pathology, Miller School of Medicine, University of Miami, Miami, FL, United States

**Keywords:** *Steno bredanensis*, rough-toothed dolphin, mass stranding, pathology, Florida, USA

## Abstract

On March 2, 2005 ~70 rough-toothed dolphins (*Steno bredanensis*) mass stranded along mud flats and associated canals on the Atlantic Ocean side of Marathon Key, Florida. Forty-six were necropsied and placed into two groups for analysis: Group-1 animals (*N* = 34; 65%) that died prior to medical intervention and rehabilitative efforts and Group-2 animals (*N* = 12; 35%) that died in rehabilitation. Thirty-four animals were females (18 adults, 5 juvenile/subadult, 7 calves, and 4 of undetermined age) and 12 were males (6 adults, 4 juvenile/subadults, 1 calf, and 1 of undetermined age). Body condition overall was fair to good in Group-1 and fair to poor in Group-2. Lesions were observed in multiple body systems. Greater than 90% of animals in both groups had respiratory lesions. Verminous sinusitis and bronchopneumonia were 2–3 times more prevalent in Group-2. Capture/exertional rhabdomyolysis was observed in Group-2 (42%). Vacuolar hepatopathies were observed in both groups including hepatic lipidosis (Group-1) and mixed etiologies (Group-2). Pancreatic and gastrointestinal tract pathologies were prevalent in Group-2 animals 56 and 75%, respectively, and included gastritis, gastric ulceration, enterocolitis, pancreatic atrophy, and pancreatitis related to physiologic stress. Group-2 more frequently had evidence of hemorrhagic diathesis present which included increased extramedullary hematopoiesis in various organs, increased hemosiderosis, and hemorrhage and hemorrhagic drainage in various organs. Central nervous system disease, primarily edema, and mild inflammation were equally prevalent. Renal proteinuria, tubular necrosis, and pigmentary deposition were observed in Group-2. Dental attrition was observed in ~40% of the groups. Gammaherpesviral-associated pharyngeal plaques were observed in 46 and 54% of Group-1 and 2 animals, respectively. Other lesions observed were mild and incidental with a frequency rate <20%. The findings from this *Steno* stranding provide a unique window into baseline individual and population clinical conditions and additional perspective into potential clinical sequelae of rehabilitation efforts.

## Introduction

**Figure 1:**
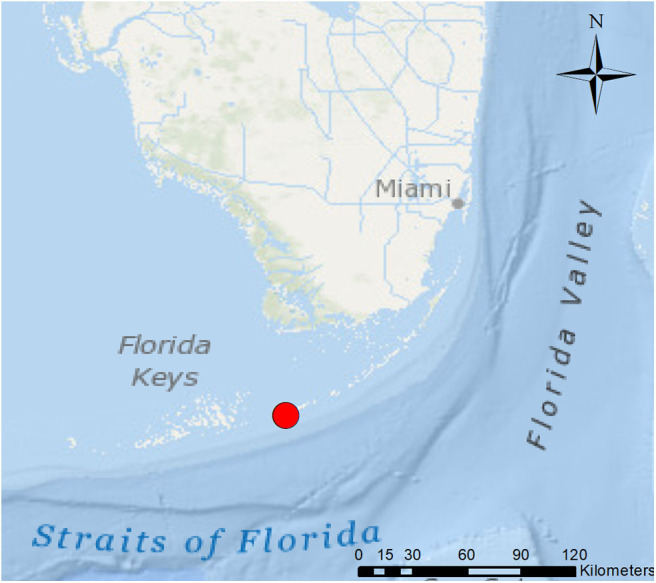
March 2, 2005 *Steno bredanensis* live mass stranding location on Atlantic Ocean side of Marathon Key, Florida (red circle; 24.71317N, −81.0535W). ^*^Frontiers Media SA remains neutral with regard to jurisdictional claims in published maps and institutional affiliations.

On March 2, 2005 ~70 rough-toothed dolphins (*Steno bredanensis*) stranded in mass along the mud flats and associated canals on the Atlantic Ocean side of Marathon Key, Florida (24.71317N, −81.0535W; Figure 1). Of the 71 cetaceans, 46 died or were euthanized and were necropsied including 2 aborted fetuses. The macroscopic (i.e., gross) and histopathologic findings from the 46 rough-toothed dolphins examined are summarized.

Investigating the stranding event was complex in that animal mortalities occurred in two phases: prior to intervention and during the course of rehabilitation for surviving animals, resulting in variation in the diseases observed. Animals were placed into two groups for analysis of findings: Group-1 (March 2nd−8th), those which died initially prior to medical intervention and rehabilitative efforts and Group-2 (March 9th–July 18th, 2005), those which died or were euthanized in rehabilitation. Group-1 has 34 animals (74%; 34/46) which were either euthanized or died spontaneously within the first 6 days of the stranding event, including two aborted premature fetuses, and are described below. Group-2 has 12 animals (26%; 12/46) which were held in rehabilitation and received nutritional support, antimicrobial, anthelmintic, and various supportive therapies for different maladies. Ultimately, 11 animals were successfully rehabilitated and released into the wild while one calf was deemed non-releasable and transferred to a marine park. No immediate cause of the stranding event was determined.

## Materials and Methods

Forty-six of the 71 animals examined were fresh dead to mildly decomposed, 13 were moderately decomposed and too autolyzed for histologic examination. Stranding data (i.e., Level A) were collected and recorded on standardized marine mammal stranding reporting forms by members of the Southeastern United States (SEUS) Marine Mammal Stranding Network according to established protocols (1). In general, stranding data consists of a unique individual identifier, reporting agency, genus and species, stranding location latitude and longitude, date observed and examined, body condition, total length, gender, and body disposition. Necropsies preformed on Group-1 animals were conducted at the Marathon Turtle Hospital (Marathon Key, FL), Harbor Branch Oceanographic Institute (HBOI) (FT. Pierce, FL) and Florida's Fish & Wildlife Research Institute (FWRI) (St. Petersburg, FL) while all Group-2 animals were necropsied at the National Marine Fisheries Service (NMFS), Miami Laboratory (Miami, FL). Necropsy notes/data were collected on a systemic and organ basis; form composition differed based on the necropsy team preferences.

There were four necropsy teams due to the large number of animals involved, protracted length of time from the stranding event to rehabilitation, and logistical challenges. Necropsy teams were composed of veterinary pathologists, clinical veterinarians, anatomists, and biologist. Typically, a standard set of tissues from all body systems were sampled, fixed in 10% neutral buffered formalin, sub-sectioned, embedded in paraffin, sectioned at 5 μm and stained with hematoxylin and eosin (HE) for routine light microscopic evaluation.

Histopathologic examination was completed by several co-author pathologists. For this reason, there was variation in recording of data and interpretation of gross and histologic findings; therefore, gross (macroscopic) and microscopic findings were combined for congruency. The macroscopic and histopathologic data were compiled to determine the prevalence of lesions in the organ systems of the stranded dolphins based on the number of samples available for any particular organ system examined.

The occurrence of an abnormal finding either solely by gross or microscopic examination resulted in a positive finding, while if the organ was without significant findings or not remarkable (NR), the result was a negative (i.e., normal finding). However, if an organ was found NR grossly and was not evaluated microscopically, the examination was incomplete and the result was considered an inconclusive finding and these animals were excluded from the general organ system prevalence by group. These animals were included in the combined gross and microscopic overall population prevalence for each organ system examined. Lesion prevalences were determined by occurrence and assessed based on severity. The significance was based on the group (overall population) and the individual (clinical significance). Significant lesions could result in high morbidity or mortality or is infectious (e.g., herpesvirus). An incidental finding was generally considered a relatively benign disease (e.g., benign neoplasm). Incidental findings could also occur irrespective of the primary cause of death or a fulminant disease process. In evaluating lesion prevalence, a finding was considered common if it was observed in 20% or greater of animals in the group; for brevity, only commonly occurring conditions will be discussed. Findings occurring at a lower prevalence (i.e., less frequently; <20%) were not considered significant to the population overall, although possibly clinically significant to the affected individual. Results are summarized by macroscopic and microscopic findings based on body system.

Microbial samples were collected using aseptic techniques and placed in transport medium (i.e., SP4 mycoplasma medium containing 20% fetal calf serum and both glucose and arginine, without antibiotics). Specimens were plated for colony growth upon receipt by the laboratory. After culturing, classical diagnostic microbiology techniques were employed including coagulase testing, streptococcus grouping reagents, specific tubed media tests, and the BBL/BS™ Crystal™ Identification System (Becton, Dickinson and Company, Franklin Lakes, NJ, USA). Samples acquired later were subjected to the Biolog^®^ bacterial identification system (BIOLOG, Hayward, CA, USA), which was used for rapid identification of many of the bacteria and fungi isolated from these animals. Two isolates of a *Mycobacterium* species obtained from a single animal were sent to the National Veterinary Services Laboratory at Ames, Iowa for speciation.

Total DNA was extracted from lesions and tissues using the DNeasy Tissue Kit (Qiagen Inc., Valencia, CA), following the manufacturer's protocol. A polymerase chain reaction (PCR) that targets the DNA polymerase gene of members of the *Herpesviridae* family has been used extensively for the detection of herpesvirus DNA from diverse mammalian species (2, 3), including cetaceans (4), and was used in our studies. A second PCR assay that targets the glycoprotein B (gB) gene of gammaherpesvirus (5) was also performed on positive samples using primers 734s and 702s (5) in order to confirm results of the DNA polymerase PCR assay and obtain sequences from a second gene. PCR amplicons were resolved by agarose gel electrophoresis and the amplified DNA fragments of the expected sizes were extracted from the gel, purified [MinElute PCR Purification Kit (Qiagen Inc.)], and sequenced using Sanger technology in our laboratory. A real-time PCR assay was standardized using the gB gene sequences obtained from gammaherpesviruses recovered from the stranded *Steno bredanensis* dolphins, after their alignment with homologous gB gene sequences from gammaherpesviruses of *Tursiops truncatus, T. aduncus, Grampus griseus, Mesoplodon densirostris*, and *Kogia sima*. Glycoprotein B sequences from gammaherpesviruses of these species were aligned using the MegAlign function of the Lasergene software (DNASTAR Inc., Madison, WI, USA) and forward and reverse primers as well as a fluorogenic probe containing locked nucleic acids were designed using highly conserved sequences within the gB genes. Additional PCRs and reverse-transcription PCRs were used on select lesion samples to detect poxviruses (6), morbilliviruses (7), and marine vesiviruses (8).

## Results

The prevalence of macroscopic lesions from 10 organ systems including cardiovascular, respiratory, digestive, endocrine, musculoskeletal, urinary, reproductive, central nervous system, integumentary, and hemolymphatic systems are presented in Table 1. Presented in Table 2, are the prevalence of histopathologic lesions from eight organ systems including respiratory, digestive, endocrine, urinary, reproductive, central nervous system, integumentary, and hemolymphatic systems.

**Table 1:** Macroscopic findings and lesion prevalence from necropsied rough-toothed dolphins.

	**Group-1**			**Group-2**		
**System and tissue**	Number examined^*^	**Lesions**	**%**	Number examined^*^	**Lesions**	**%**
Cardiovascular system	32			2		
Myocardial fibrosis		0	0		1	**50.0**
Arteriosclerosis		1	3.1		1	**50.0**
Aneurysm		0	0		1	**50.0**
Thrombosis		0	0		1	**50.0**
Respiratory system and sinuses	30			12		
Pterygoid sinusitis nasitremiasis		0	0.0		5	**41.7**
Pulmonary congestion/hemorrhage		12	**40.0**		3	**25.0**
Bronchopneumonia		1	3.3		6	**50.0**
Pleural fibrosis		2	6.7		3	**25.0**
Oronasal pharynx	3			6		
Pharyngitis		0	0.0		2	**33.3**
Mucosal plaque		2	**66.7**		4	**66.7**
Nervous system	18			11		
Meningeal hemorrhage		4	**22.2**		2	18.2
Hemolymphatic system						
Lymph nodes	32			12		
Lymphadenopathy		17	**53.1**		7	**58.3**
Depletion		0	0.0		3	**25.0**
Edema		2	6.3		3	**25.0**
Pigment deposition		0	0.0		5	**41.7**
Spleen	26			12		
Congestion		6	**23.1**		0	0.0
Thymus	8			3		
Thymic involution		1	12.5		1	**33.3**
Edema		0	0.0		2	**66.7**
LALT	2			11		
Laryngitis		0	0.0		8	**72.7**
Hyperplasia		1	**50.0**		6	**54.5**
Digestive system	30			11		
Gastroduodenal, trematodiasis		18	**60.0**		7	**63.6**
Fundic stomach, gastritis		0	0.0		4	**36.4**
Fundic stomach, ulceration		1	3.3		3	**27.3**
Fundic stomach, Hemorrhage		1	3.3		4	**36.4**
Pyloric stomach, Hemorrhage		0	0.0		2	**18.2**
Enteric, hemorrhage		1	3.3		6	**54.5**
Enteritis		1	3.3		3	**27.3**
Enterocolitis		0	0.0		4	**36.4**
Oral cavity	10			12		
Dental attrition		4	**40.0**		5	**41.7**
Tooth fracture		2	**20.0**		0	0.0
Glossitis		0	0.0		4	**33.3**
Tongue, ulceration		2	**20.0**		4	**33.3**
Ulcerative stomatitis		2	**20.0**		0	0.0
Pancreas	32			11		
Pancreatitis		1	3.1		6	**54.5**
Necrosis		2	6.3		6	**54.5**
Hemorrhage		2	6.3		6	**54.5**
Saponification		1	3.1		3	**27.3**
Hepatobiliary system	32			11		
Hepatic trematodiasis					3	**27.3**
Congestion		14	**43.8**		0	0.0
Musculoskeletal system	34			12		
Scoliosis/muscle contracture		1	2.9		3	**25.0**
Muscle necrosis/pallor		0	0.0		4	**33.3**
Rhabdomyolysis		1	2.9		5	**41.7**
Endocrine system						
Adrenal gland	30			12		
Adrenal gland cortical hyperplasia		7	**23.3**		3	**25.0**
Congestion		7	**23.3**		4	**33.3**
Thyroid gland	19			12		0.0
Thyroglossal cyst		1	5.3		4	**33.3**
Urinary system						
Kidney	32			12		
Interenicular fat serous atrophy		7	**21.9**		0	0.0
Urinary bladder	32			12		
Proteinuria		NA	NA		3	**25.0**
Hematuria		NA	NA		3	**25.0**
Reproductive system	32			12		
Female	25			9		
Leiomyoma		1	4.0		2	**22.2**
Endometritis		0	0.0		2	**22.2**
Cervicitis		0	0.0		2	**22.2**
Milliary cervicitis		0	0.0		2	**22.2**
Endometrial adenocarcinoma		0	0.0		2	**22.2**
Body as a whole, body cavity						
Emaciation/poor condition		4	12.5		6	**50.0**
Thin/fair condition		9	**28.1**		2	16.7
NRG/good condition		21	**65.6**		4	**33.3**
Abdominal serosanguinous effusion		4	12.5		5	**41.7**
Thoracic serosanguinous effusion		0	0.0		3	**25.0**
Pericardial serosanguinous effusion		0	0.0		9	**75.0**
Integumentary system	18			12		
Abrasion		5	**27.8**		2	16.7
Laceration		4	**22.2**		1	8.3
Fibrosis		4	**22.2**		2	16.7
Dermatitis		1	5.6		6	**50.0**
Pox dermatitis		3	16.7		3	**25.0**

* *The denominator for prevalence determination represents the number of samples available for any particular organ system examined. Light gray indicates sex and organ system headings. Bold numbers indicates findings in >20% of the samples examined for either or both groups. NA, not applicable*.

**Table 2:** Microscopic findings and lesion prevalence from necropsied rough-toothed dolphins.

	**Group-1**			**Group-2**		
**System and Tissue**	Number Examined^*^	**Lesions**	**%**	Number Examined^*^	**Lesions**	**%**
Respiratory system and sinuses	27			12		
Pterygoid sinusitis nasitremiasis		0	0.0		5	**41.7**
Pulmonary congestion/hemorrhage		4	14.8		6	**50.0**
Pulmonary edema		14	**51.9**		1	8.3
Bronchopneumonia		6	**22.2**		7	**58.3**
Pneumonitis		1	3.7		4	**33.3**
Extramedullary hematopoiesis		1	3.7		3	**25.0**
Oronasal pharynx	3			10		
Nasopharyngitis		0	0.0		6	**60.0**
Pharyngitis		3	**100.0**		0	0.0
Cytopathic changes		2	**66.7**		5	**50.0**
Hemorrhage		0	0.0		3	**30.0**
Scirrhous ductal carcinoma		0	0.0		1	10.0
Epithelial hyperplasia/acanthosis		2	**66.7**		4	**40.0**
LALT	2			11		
Laryngitis		1	**50.0**		11	**100.0**
Gland abscessation		0	0.0		8	**72.7**
Follicular hyperplasia		2	**100.0**		3	**27.3**
Glandular hyperplasia		1	**50.0**		3	**27.3**
Hemorrhage		1	**50.0**		2	18.2
Trematode eggs		0	0.0		4	**36.4**
Nervous system	23			12		
Anoxic change		4	17.4		7	**58.3**
Purkijne cell drop-out		1	4.3		3	**25.0**
Neuronal satellitosis		2	8.7		8	**66.7**
Microglial nodules		1	4.3		3	**25.0**
Gliosis		4	17.4		7	**58.3**
Myelin hyalinosis		1	4.3		3	**25.0**
Myelin vacuolation		2	8.7		7	**58.3**
Demyelination		7	**30.4**		2	16.7
Axonal degeneration/spheroids		4	17.4		3	**25.0**
Neuropil vacuolation (spongiosis, edema)		6	**26.1**		9	**75.0**
Congestion		0	0.0		3	**25.0**
Perivascular edema		9	**39.1**		9	**75.0**
Perivascular hemorrhage		5	**21.7**		3	**25.0**
Perivasculitis non-suppurative		5	**21.7**		1	8.3
Siderophages/hemosiderosis		3	13.0		3	**25.0**
Pigment deposition		1	4.3		3	**25.0**
Non-suppurative meningitis		2	8.7		5	**41.7**
Lafora bodies (polyglucosan bodies)		1	4.3		4	**33.3**
Meningeal edema		2	8.7		5	**41.7**
Meningeal hemorrhage		3	13.0		9	**75.0**
Meningeal fibrosis		1	4.3		7	**58.3**
Choroid plexus, edema		0	0.0		3	**25.0**
Hemolymphatic system						
Lymph nodes	24			12		
Hyperplasia		6	**25.0**		3	**25.0**
Depletion/hypocellularity		3	12.5		10	**83.3**
Hemorrhage		0	0.0		5	**41.7**
Inflammation drainage		4	16.7		12	**100.0**
Hemorrhage drainage		2	8.3		10	**83.3**
Lymphadenitis		1	4.2		7	**58.3**
Reactive change		4	16.7		10	**83.3**
Histiocytosis		4	16.7		9	**75.0**
Squamous metaplasia		0	0.0		1	9.1
Plasmacytosis		2	8.3		3	**25.0**
Extramedullary hematopoiesis		1	4.2		4	**33.3**
Pigment deposition/hemosiderosis		5	**20.8**		8	**66.7**
Pigment deposition/hematin		0	0.0		4	**33.3**
Pigment deposition/anthracosis		6	**25.0**		3	**25.0**
Spleen	19			12		
Splenitis		1	5.3		3	**25.0**
Hemorrhage		3	15.8		4	**33.3**
Pigment deposition/hemosiderosis		2	10.5		3	**25.0**
Extramedullary Hematopoiesis		1	5.3		8	**66.7**
Lymphoid depletion		1	5.3		6	**50.0**
PAL hyalinosis		0	0.0		4	**33.3**
Thymus	14			6		
Thymic Involution		5	**35.7**		1	16.7
Lymphoid depletion/atrophy		2	14.3		5	**83.3**
Hemorrhage		2	14.3		4	**66.7**
Edema		2	14.3		4	**66.7**
Pancreas	12			11		
Zymogen granule depletion		3	**25.0**		8	**72.7**
Necrosis		4	**33.3**		6	**54.5**
Saponification/lipolysis		1	8.3		3	**27.3**
Ductitis		1	8.3		4	**36.4**
Duct hyperplasia		2	16.7		3	**27.3**
Hemorrhage		4	**33.3**		7	**63.6**
Islet of Langerhan eosinophilic intranuclear inclusions		2	16.7		6	**54.5**
Hepatobiliary system	26			11		
Vacuolar change		0	0.0		5	**45.5**
Hydropic change		0	0.0		3	**27.3**
Fatty change		6	**23.1**		0	0.0
Hemosiderosis (iron deposition)		6	**23.1**		7	**63.6**
Hematin deposition (trematode pigment)		2	7.7		3	**27.3**
Bacterial emboli		1	3.8		3	**27.3**
Cholangitis/portal hepatitis		7	**26.9**		5	**45.5**
Cholangiohepatitis		0	0.0		5	**45.5**
Cholestasis		0	0.0		3	**27.3**
Bile duct hyperplasia		3	11.5		5	**45.5**
Fibrosis		1	3.8		4	**36.4**
Central vein fibrosis		3	11.5		4	**36.4**
Congestion		9	**34.6**		5	**45.5**
Extramedullary hematopoiesis		3	11.5		3	**27.3**
Cholestasis		0	0.0		3	**27.3**
Endocrine system						
Pituitary gland	5			11		
Rathke's pouch cyst		1	**20.0**		0	0.0
Adenohypophysis, cyst		1	**20.0**		3	**27.3**
Adenohypophysis, thyroidization		1	**20.0**		11	**100.0**
Adenohypophysis, mineralization		0	0.0		3	**27.3**
Hemorrhage		1	**20.0**		0	0.0
Infundibular stalk, congestion		0	0.0		3	**27.3**
Adrenal gland	21			12		
Adrenal gland, cortical hyperplasia		5	**23.8**		3	**25.0**
Extramedullary hematopoiesis		2	9.5		5	**41.7**
Adrenalitis		0	0.0		6	**50.0**
Necrotizing adrenalitis		0	0.0		4	**33.3**
Fibrosis		0	0.0		3	**25.0**
Hemorrhage		1	4.8		3	**25.0**
Thyroid gland	15			12		
Thyroid gland, follicular cysts		5	**33.3**		3	**25.0**
Thyroid gland, thyroglossal cyst		0	0.0		3	**25.0**
Papillary hyperplasia		0	0.0		3	**25.0**
Colloid depletion/drop out		4	**26.7**		6	**50.0**
Hemosiderin deposition		2	13.3		5	**41.7**
Congestion		2	13.3		8	**66.7**
Urinary system						
Kidney	27			12		
Nephritis		4	14.8		5	**41.7**
Interstitial fibrosis		3	11.1		4	**33.3**
Tubular pigment deposition		0	0.0		6	**50.0**
Glomerulopathy (membranous)/Bowman's hyalinization		6	**22.2**		5	**41.7**
Nephrosis/tubular necrosis		1	3.7		4	**33.3**
Medullary mineralization		4	14.8		7	**58.3**
Reproductive system	17			12		
Female	11			9		
Leiomyoma		1	9.1		2	**22.2**
Endometritis		7	**63.6**		2	**22.2**
Uterine angiopathy (sclerosis)		4	**36.4**		0	0.0
Cervicitis		0	0.0		2	**22.2**
Pseudocervicitis		0	0.0		5	**55.6**
Cervix, squamous metaplasia		0	0.0		2	**22.2**
Endometrial Adenocarcinoma		0	0.0		2	**22.2**
Mammary gland	13			7		
Mastitis		5	**38.5**		2	**28.6**
Benign adenoma		0	0.0		1	11.1
Male	6			3		
Epididymitis		0	0.0		1	**33.3**
Epididymis, luminal hemorrhage		0	0.0		1	**33.3**
Integumentary system	14			6		
Atrophy		5	**35.7**		4	**66.7**
Dermatitis		1	7.1		6	**100**

* *The denominator for prevalence determination represents the number of samples available for any particular organ system examined. Light gray indicates sex and organ system headings. Bold numbers indicates findings in >20% of the samples examined for either or both groups*.

### Body as a Whole and Integumentary System

The overall body condition of the animals from both groups presented for post-mortem examination were generally (>50%) in good to fair (thin) condition with adequate blubber layers and good muscling such that skeletal protuberances were not readily apparent. In Group-1, 66% (21/32) animals were in good condition and 28% (9/32) were fair. However, in Group-2 50% (6/12) animals were in poor condition (i.e., emaciated), characterized by post-nuchal fat pad softening, sunken cervical region, and prominent vertebral transverse process etc. while 33% (4/12) were in good condition and 17% (2/12) were fair. In addition, grossly hypodermal atrophy was observed in fewer Group-1 animals (11%; 2/18) vs. in Group-2 (33.3%; 4/12). Although, microscopically, hypodermal adipose tissue atrophy was observed commonly in both Group-1 (35.7%; 5/14) and in Group-2 (66.7%; 4/6). In Group-1 animals, superficial cutaneous abrasions (27.8%; 5/18) and lacerations (22.2%; 4/18) predominated. In Group-2, both of these lesions occurred in <10% of animals. Conversely, secondarily infected traumatic dermal inflammation was more predominant in Group-2 animals (50%; 6/12) vs. Group-1 (5.6%; 1/18). The rostrum and distal extremities (e.g., caudal tail and pectoral fins) were primarily involved, with lesions characterized by locally extensive areas of epidermal ulceration and mixed dermal inflammation. The lesions varied in severity and chronicity between animals depending on the presence of secondary intralesional organisms (e.g., bacterial colonies) and vascular involvement consisting of perivasculitis, vasculitis, and vascular thrombosis. An individual from each group presented with proliferative pyogranulomatous dermatitis containing intralesional ciliated protozoa, suggestive of *Kyaroikeus cetarius*. Gross dermal changes consistent with Dolphin-poxvirus infection were observed in 25% of Group-2 animals and less commonly in Group-1 (16.7%; 3/18).

### Respiratory System and Sinuses

The overall prevalence of lesions in the respiratory system was 87% (40/46) and consisted of findings in the cranial sinuses, larynx, trachea, or lungs. Four animals from Group-1 had no gross changes observed involving any component of the respiratory system, were not examined microscopically, and were therefore considered to have inconclusive findings (12%; 4/34). Two fetuses, which had no microscopic lesions present, were also not included.

In Group-1, the respiratory system lesion prevalence was 93% (28/30) with an abnormality found involving at least one component of the respiratory system; in Group-2, the respiratory system lesion prevalence was 100% (12/12). In Group-1, cranial sinus verminous sinusitis was observed in 13% (4/30) of animals with three animals having nematodes consistent with *Stenurus* sp., while no parasite species was identified in one animal. In Group-2, verminous sinusitis was observed in 50% (6/12) of animals of which trematodes consistent with *Nasitrema* sp. were in 42% (5/12) of cases and *Stenurus* sp. in 8% (1/12).

Grossly, pulmonary congestion with or without hemorrhage was observed in 40% (12/30) of Group-1 animals and 25% (3/12) of Group-2 animals. Microscopically, this change was appreciated less frequently in Group-1 animals, occurring in 15% (4/27), while in Group-2 animals these changes were observed in 50% (6/12). Microscopically, in Group-1, common findings included pulmonary edema (52%; 14/27) and bronchopneumonia (22%; 6/27) while grossly these changes were rarely observed. Clinically significant bacterial and /or fungal suppurative to pyogranulomatous pneumonia was observed in 38% (3/8) of Group-1 animals.

Respiratory changes for which there were concurrent culture results included: pyogranulomatous pneumonia with abscessation and intralesional filamentous fungi associated with a mixed growth of *Vibrio alginolyticus, Enterobacter cloacae*, and *Brevibacillus brevis*; pyogranulomatous bronchointerstitial pleuropneumonia associated with a mixed growth of *Staphylococcus xylosis* and *Carnobacterium piscicola*; and a suppurative and edematous bronchopneumonia associated with a pure growth of *Vibrio* (formerly *Photobacterium*) *damselae*. The filamentous fungal hyphae observed microscopically in the lung were not isolated, however, were morphologically suggestive of an oomycetes.

In Group-2, inflammation of the laryngeal associated lymphoglandular tissue (LALT) was observed in all animals. Other common microscopic findings in Group-2 included bronchopneumonia (58%; 7/12); pulmonary congestion/hemorrhage and pleural fibrosis (50%; 6/12); pneumonitis, pleural edema, pleuritis (33%; 4/12); and extramedullary hematopoiesis (25%; 3/12). Clinically significant bacterial and/or fungal, suppurative to pyogranulomatous pneumonia was observed in 45% (5/11) of respiratory lesions. Respiratory changes for which there were concurrent culture results included: necrosuppurative bronchopneumonia with a pure growth of *Citrobacter freundii* which was also isolated from the pleura and the larynx in a mixed culture with *Pseudomonas pseudoalcaligines* and *Escherichia coli*, and *Enterococcus faecalis*, respectively; suppurative bronchopneumonia associated with a mixed growth of *E. coli, Shewanella putrefaciens, Enterococcus mundtii*, and *Carnobacterium divergens*; necrosuppurative and fibrosing bronchopneumonia with abscessation associated with a mixed growth of *E. coli, Klebsiella pneumoniae, E. faecalis*, and *Streptococcus anginosis* along with laryngeal isolates of *E. faecalis, Vibrio metschnikovii, C. freundii, Aeromonas veroni*, and a *Vibrio* sp.; necrosuppurative and edematous bronchopneumonia associated with a mixed growth of *Staph. xylosis, Arcanobacterium* (formerly *Actinomyces* sp.) *pyogenes* and an unidentifiable coryneform gram positive bacillus (Supplementary Figure 1); and lastly a pyogranulomatous bronchopneumonia with microscopic fungal hyphae associated with a mixed growth of *Morganella morganii, E. coli, Staph. aureus*, and *Enterococcus gallinarum*. Fungi observed microscopically were not isolated from the lung; however, were grown from the pharynx (*Penicillium brevicompactum*) mixed with *M. morganii* and *Staph. aureus*. Interestingly, for this animal similar fungal hyphae observed microscopically in the brain were also not cultured (see Central Nervous System).

### Digestive System

The overall lesion prevalence rate for both groups was 91% (42/46) which included either gross or microscopic findings in the alimentary tract (i.e., mouth to anus), liver and/or pancreas.

Four animals from Group-1 had inconclusive findings (12%), while no animal was found to be normal. In both groups, macroscopic digestive system lesion prevalence was 100% (Group-1, 30/30; Group-2, 12/12). Tooth wear and loss (dental attrition) was observed grossly in a total of nine (41%; 9/22) animals from both groups combined (Group-1, 4/10; 40% and Group-2, 5/12; 42%). In some animals, the condition was extreme in that no teeth were recovered for age determination.

**Figure 2:**
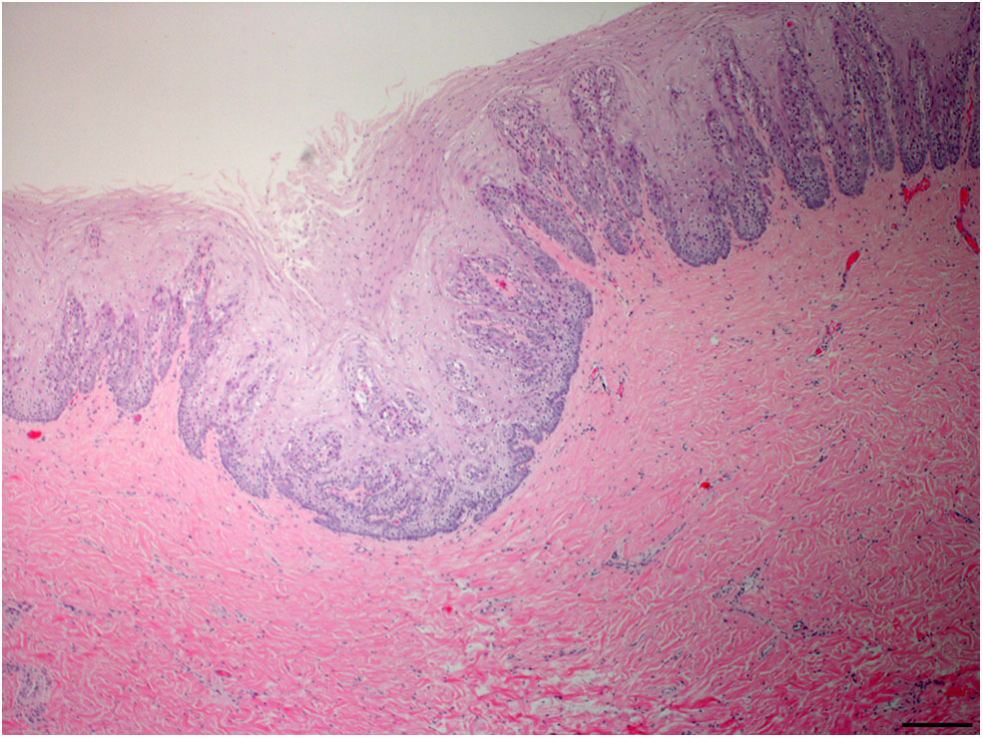
Oropharyngeal mucosal plaque, rough-toothed dolphin (case R305). Submucosa, focal invagination of hyperplastic epithelium with moderate retention of stratum externum layer, and thickening of the stratum intermedium with epithelial peg disorganization and fusion (HE) Obj. 4x. Bar = 200 μm.

**Figure 3:**
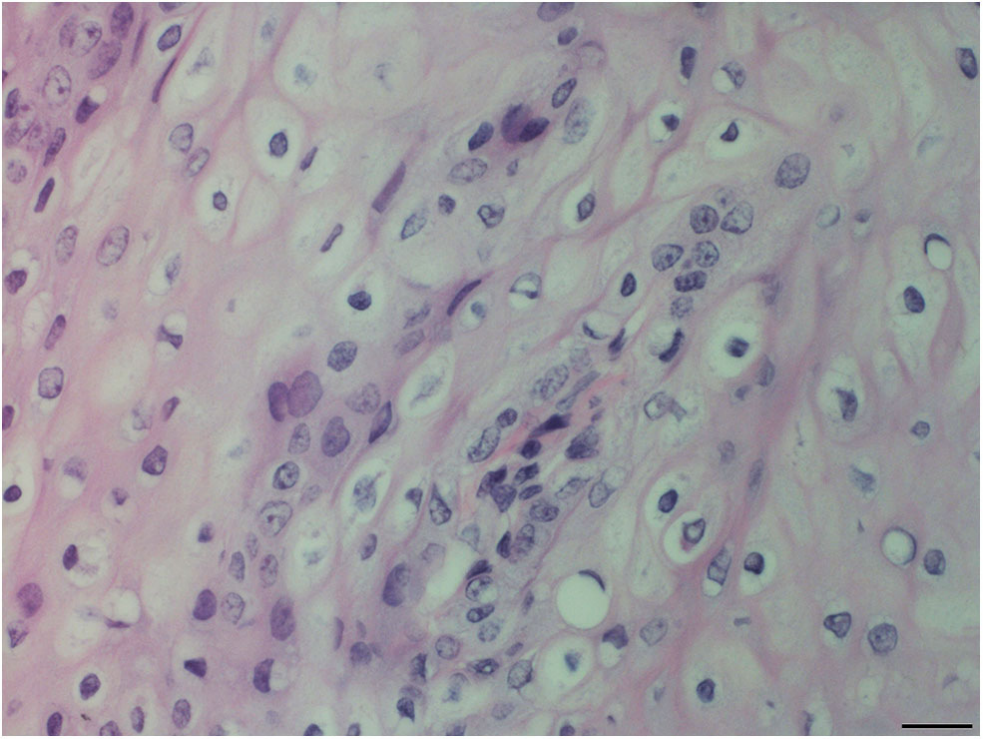
Oropharyngeal mucosal plaque, rough-toothed dolphin (case R305). Hyperplastic epithelial cells with pale foamy cytoplasm. Epithelial cells have clear perinuclear cytoplasmic halos or vacuolation with pyknotic or hyperchromatic displaced nuclei (HE). Obj. 40x. Bar = 20 μm.

**Figure 4:**
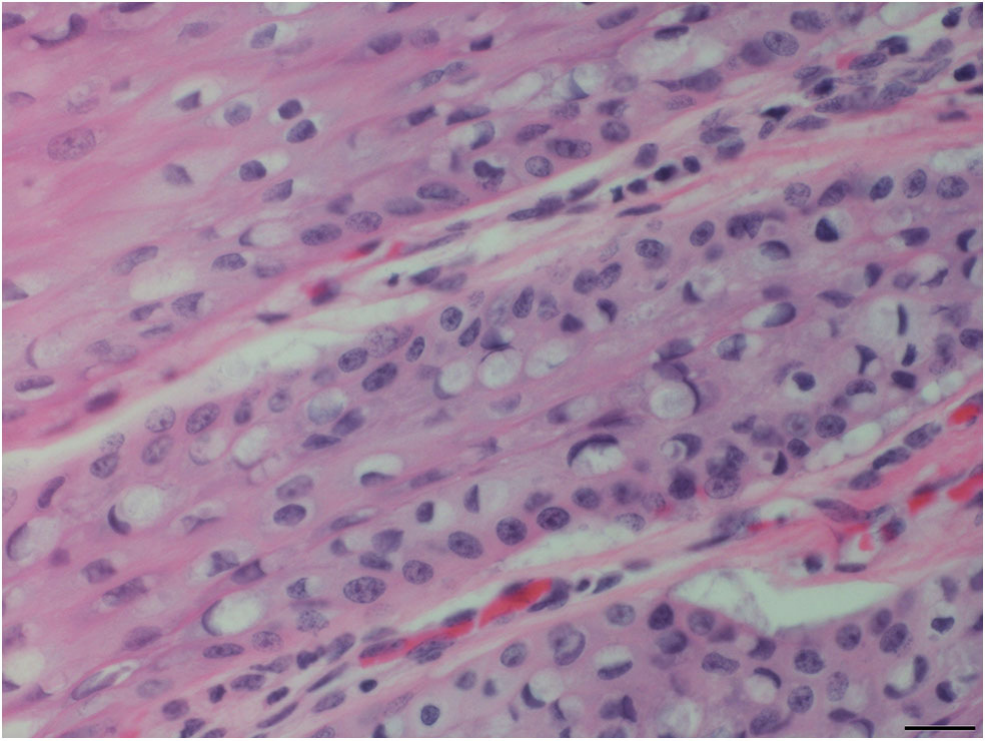
Tongue mucosal plaque, rough-toothed dolphin (case R305). Suprabasilar epithelial cells have perinuclear cytoplasmic vacuolation, with hyperchromatic marginated nuclei and amphophilic intracytoplasmic inclusion bodies (HE). Obj. 40x. Bar = 20 μm.

Pharyngeal mucosal plaques were observed grossly in a total of six animals from both groups combined (Group-1, 2/3; 67% and Group-2, 4/6; 67%) and on the epiglottis of an individual (Supplementary Figure 2). Microscopically, pharyngeal epithelial hyperplasia (i.e., acanthosis) and cytopathic changes including epithelial cells with koilocyte-like features consisting of clear perinuclear cytoplasmic halos, perinuclear cytoplasmic vacuolation, anisokaryosis, nuclear pyknosis, and displaced hyperchromatic paracentric nuclei were observed. Together these and other features like amphophilic intracytoplasmic inclusion bodies and occasional eosinophilic intranuclear inclusions were also observed in the pharyngeal mucosa in both groups at 46% (6/13) and 54% (7/13), respectively (Figures 2–4). Incidentally, the nasopharynx parapharyngeal skeletal muscle had a small, scirrhous ductal carcinoma which had not been observed grossly. Microscopically, the parapharyngeal skeletal muscle fascicles were displaced by a dense, well-demarcated, non-encapsulated expansile mass with multifocal tendrils of mild fascicle infiltration along the perimysium, and endomysium. The mass consisted of pleomorphic, cuboidal to flattened elongated neoplastic epithelial cells haphazardly arranged forming nests, acini, and lining irregular and abortive ducts scattered within an abundant, dense, acidophilic to myxohyaline, spindle cell stroma. Mitotic figures were not observed.

In five of the six mucosal plaque cases, both PCRs used to detect herpesvirus DNA targeting the DNA polymerase and the glycoprotein B demonstrated the presence of herpesvirus DNA in mucosal lesions of the oropharyngeal region, specifically, epiglottic and pharyngeal lesions (Table 3). Sequencing of the DNA polymerase gene fragments and their analyses using the BLAST function of the National Center for Biotechnology Information (NCBI) showed that the various sequenced DNA fragments were all identical and corresponded to the DNA polymerase gene of dolphin gammaherpesviruses, as determined by comparisons to homologous sequences from the GenBank database. The most related homologous sequences corresponded to those from pharyngeal tonsils of a *Grampus griseus* (95% identity, NCBI accession number (No.) KP995680) that stranded in Valencia, Spain; a vaginal lesion from a captive *Tursiops truncatus* (82% identity, NCBI No. AY952777) in Florida; and a *Delphinus delphis* (79% identity, NCBI No. MG437207) that stranded on the Northeast Portuguese coast. These three sequences corresponded to dolphin gammaherpesviruses within the *Herpesviridae* family of herpesviruses. The partial nucleotide sequence of the DNA polymerase gene of a *Steno bredanensis* gammaherpesvirus V1944_R356 has been deposited in the GenBank database under accession number KX424962.

**Table 3:** Presence of gammaherpesvirus DNA in tissues of rough-toothed dolphins (*Steno bredanensis*) that stranded in 2005 in the Gulf of Mexico, Florida.

**Dolphin**	**Pharynx lesions**	**Skin lesions**	**Spleen**	**Cerebellum, thymus**	**Kidney, adrenal**	**Cerebrum**	**Tongue, epiglottis**	**Mouth lesions**	**Pseudo- cervix, liver**	**LALT**	**Lung, pancreas**
R132	^**^			0.0/–	0.0/–	0.0/–	0.0/–		0.0/–, 0.0/–	0.0/–	0.0/–
R137			0.0/–	0.0/–	0.0/–, 0.0/–	0.0/–	0.0/–		0.0/–	0.0/–	0.0/–, 0.0/–
R303	0.0/–	0.0/–	0.0/–	0.0/–	0.0/–, 0.0/–	0.0/–	**25.9/+**		0.0/–	0.0/–	0.0/–, 0.0/–
R305	^*^20.9/+	0.0/–	0.0/–	n/a	0.0/	0.0/–				0.0/–	0.0/–, 0.0/–
R308	**18.2/+**		0.0/–	0.0/–	0.0/–	0.0/–	0.0/–, 0.0/–	0.0/–	0.0/–, 0.0/–	0.0/–	
R352		0.0/–			0.0/–	0.0/–	0.0/–				
R356	**16.5/+**		0.0/–			0.0/–				0.0/–	0.0/–, 0.0/–
R363			0.0/–	0.0/–	0.0/–	0.0/–				0.0/–	0.0/–
Y365	**17.6/+**		0.0/–	0.0/–, 0.0/–	0.0/–, 0.0/–	0.0/–			0.0/–	0.0/–	0.0/–
Y302			0.0/–	0.0/–	0.0/–	0.0/–		0.0/–	0.0/–, 0.0/–	0.0/–	0.0/–, 0.0/–
Y327			0.0/–		0.0/–	0.0/–				0.0/–	0.0/–
Y375	0.0/–		0.0/–	0.0/–	0.0/–	0.0/–			0.0/–	0.0/–	0.0/–, 0.0/–

* *Ct value of a qPCR targeting the glycoprotein B gene of cetacean gammaherpesvirus/bolded positive (+) or negative (–) result of a conventional panherpes PCR targeting the DNA polymerase gene of alpha- or gamma- cetacean herpesviruses*.

** *Empty spaces correspond to tissues not being available. LALT, Laryngeal-associated lymphoid tissue*.

All samples that yielded a positive result for the DNA polymerase gene of dolphin gammaherpesvirus also yielded positive results in both the conventional PCR and the real-time PCR that target the glycoprotein B of dolphin gammaherpesviruses. The complete glycoprotein B gene sequence was obtained from one of the *Steno bredanensis* (V1944_R356) gammaherpesviruses, sequenced and compared to homologous sequences stored in the GenBank database. The sequence had highest identity (77% identity to NCBI accession No. KX528022 and KX494869) to sequences of the glycoprotein B of gammaherpesviruses derived from skin and vaginal lesions of *Tursiops truncatus* from Florida and the Bahamas, respectively. The complete sequence of the glycoprotein B gene and its coded protein have been deposited to the GenBank database of the NCBI accession No. MT038045. There was no evidence of infection by poxviruses, morbilliviruses or marine vesiviruses demonstrated in any of the selected tissue and lesion samples tested for theses viral agents.

Grossly, the alimentary tract in Group-2 had the greatest number of changes which were observed in >20% of animals. In Group-2 common findings included: fundic stomach gastritis (36%; 4/11), fundic ulceration (27%; 3/11), enteritis (27%; 3/11), fundic stomach hemorrhage (36%, 4/11), enterocolitis (36%; 4/11), enteric hemorrhage (54%; 6/11), and verminous gastritis involving the various compartments. Conversely, in Group-1 pyloric stomach *Braunina* sp. infestation (i.e., Brauniniasis) was the only finding in >20% occurring in 23% (7/30) of animals while in Group-2 it was observed less commonly (9%; 1/11). In Group-1, the gastric chambers of every animal examined were devoid of food material with 47% (9/19) being empty, 42% (8/19) containing <10 g of indigestible prey item hard parts (i.e., spines, lens, and squid beaks) and two having scant amounts of plant material (i.e., sargassum). Alimentary tract changes involving the stomach and intestine were not evaluated for prevalence based on microscopic findings due to the rapid development of autolysis in these tissues which would be a major confounding factor in interpreting the results. The high prevalence of gastrointestinal inflammation and mucosal hemorrhage in Group-2 was a significant finding for the group and was clinically relevant.

**Figure 5:**
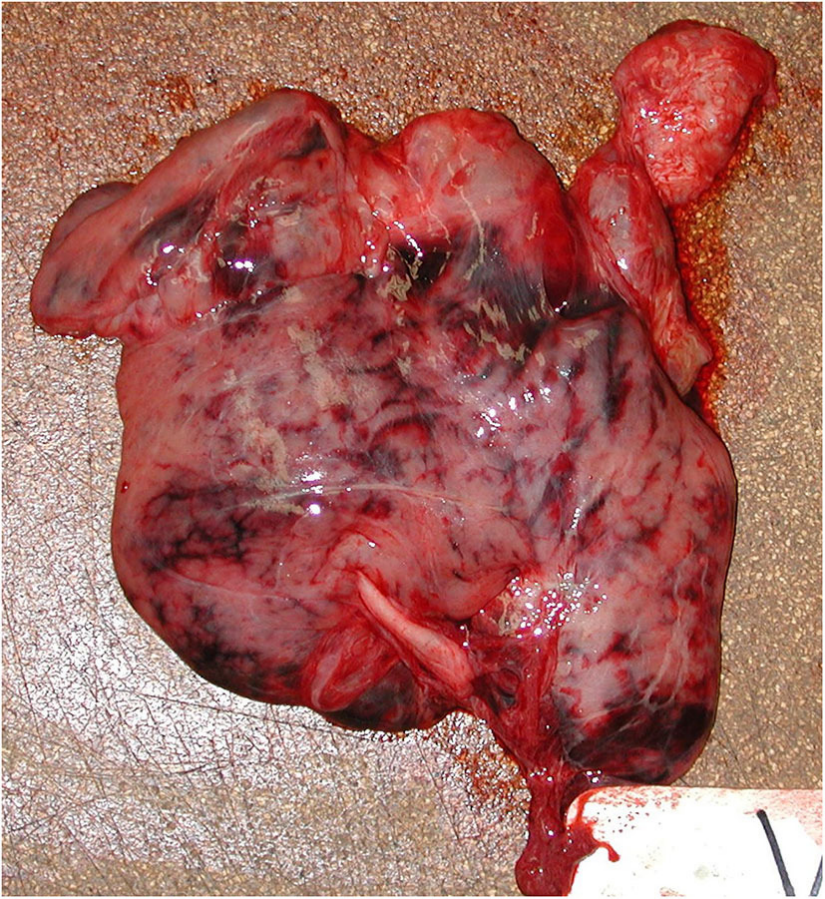
Pancreas, acute necrohemorrhagic pancreatitis, rough-toothed dolphin (case Y375). Pancreas has marked, multifocal to confluent, irregular dark red-maroon hemorrhages suffused within the parenchyma.

Grossly, acute necrohemorrhagic pancreatitis was observed in 55% (6/11) Group-2 animals (Figure 5). Microscopically, pancreatic necrosis and hemorrhage were found in 33% (4/12) of animals from Group-1, while in Group-2, 55% (6/11) had pancreatic necrosis and 64% (7/11) had pancreatic hemorrhage (Supplementary Figure 3). Pancreatic acinar cell zymogen granule depletion was found commonly in animals with or without pancreatic degenerative changes (i.e., necrosis) in Group-1 (25%; 3/12) and 73% (8/11) in Group-2. Pancreatic ductitis was found 54% (6/11) in Group-2, two cases of which were associated with trematode infestation consistent with *Campula* sp. (Group-2, 2/11; 18%).

Intrahepatic bile duct trematodiasis was seen grossly in 27% (3/11) of Group-2 animals whereas; it was not appreciated grossly in Group-1 animals and observed microscopically in only one from this group. Macroscopically, verminous gastritis involving primarily the pyloric stomach chamber due to either *Pholeter* sp. or *Braunina* sp. was a pronounced finding in 57% of Group-1 animals and 70% of Group-2 animals. In certain animals, these parasites were also observed to a lesser extent in the duodenum which in certain cases resulted in inflammation and partial occlusion of the common bile duct. Biliary cystadenomas were appreciated in both groups combined was found in 16% (5/31) overall occurring in 15% of Group-1 animals (3/20) and 18% of Group-2 animals (2/11).

In the liver, microscopic changes from both groups were predominated by degenerative changes, inflammatory/infectious causes, and changes consistent with vascular defects. Microscopically, vacuolar hepatopathy of the lipid type (i.e., fatty change) was observed in 23% (6/26) of Group-1 animals, while an unclassified vacuolar hepatopathy was observed in 46% (5/11) of Group-2 with fewer animals having hepatocellular hydropic degeneration. The elevated prevalence of hepatic fatty change in Group-1 was clinically significant in that this change, while generally a common non-specific change, could be indicative of fat mobilization from stores around the body (i.e., perirenal fat, intracoronary groove, mesenteric fat, etc.) and accumulation within hepatocytes across the group. In Group-2, vacuolar hepatopathy may represent a similar process in these animals; however, the change was interpreted broadly such that other causes of vacuolar change could be considered as differentials (e.g., hypoxia, toxemia etc.). Sinusoidal and periportal macrophage (Kupffer cell) accumulation of hemosiderin (i.e., hemosiderosis) was observed in >20% of animals in both groups with animals in Group-2 having a higher prevalence (64%; 7/11). In Group-2, sites of extramedullary hematopoiesis were observed in 27% (3/11) of animals while it was observed at a reduced rate in Group-1 (12%; 3/26). Hemorrhagic diathesis, as evidenced by sites of hemosiderosis and the need for increased hematopoiesis with the establishment of sites of extramedullary hematopoiesis, was a significant finding for Group-2.

### Hemolymphatic System

The total stranded population had an overall lesion prevalence rate of 83% (38/46) in the hemolymphatic system involving the thymus, spleen and various lymph nodes, on combined gross and microscopic examination. Bone marrow was not evaluated. In Group-1, four animals had inconclusive findings (12%; 4/34) and the two fetuses had findings consistent with fetal development, these animals were therefore excluded from the microscopic analysis. In Group-1 and Group-2, the hemolymphatic system lesion prevalence rate was 76% (26/34) and 100% (12/12), respectively.

Various lymph nodes were examined; in Group-2, inflammation drainage was observed in at least one lymph node from every animal examined (100%; 12/12). Other common microscopic findings in Group-2 included: lymphoid depletion, hemorrhage drainage, and reactive change (83%; 10/12); histiocytosis (75%; 9/12); hemosiderosis (67%; 8/12); lymphadenitis (58%; 7/12); extramedullary hematopoiesis (EMH) and hematin (i.e., fluke pigment) deposition in macrophages (33%; 4/12); and lymphoid hyperplasia, plasmacytosis, and anthracosis (25%; 3/12). In Group- 2, *Mycobacterium mucogenicum* was cultured as a pure isolate from prescapular lymph node with granulomatous lymphadenitis and was also isolated from an exudative ureter suggesting a systemic infection in that animal. In Group-1, common microscopic findings included: lymphoid hyperplasia and anthracosis (25%; 6/24) and hemosiderosis (21%; 5/24). Comparatively, the majority of common changes observed in Group-2 were also observed in Group-1 but in fewer animals at a reduced frequency.

Microscopically, the spleen and thymus had fewer changes than the lymph nodes for both groups. In Group-1, splenic changes observed were not present in >20% of the animals examined; in contrast, common splenic changes in Group-2 included: EMH (67%; 8/12); lymphoid depletion (50%; 6/12); periarterial lymphatic sheath (PAL) hyalinosis and hemorrhage (33%; 4/12); and hemosiderosis (25%; 3/12). The common thymic change in Group-1 was thymic lymphoid involution (36%; 5/14) which was present in 17% (1/6**)** of Group-2 animals.

However, in Group-2 thymic lymphoid depletion was observed in 83% (5/6) with hemorrhage and edema both in 67% (4/6). Combined, thymic involution microscopically was observed in 30% (6/20) of animals from both groups.

### Endocrine System

The overall lesion prevalence rate in the endocrine system including the adrenal, thyroid and pituitary glands for both groups on gross and microscopic examination was 80% (35/44). Eight animals from Group-1 were not examined microscopically and had inconclusive findings (25%; 8/32) while only one animal was found to be without a significant finding by both gross and microscopic examination in the endocrine system (i.e., normal). By groups, the endocrine system lesion prevalence was 96% (23/24) in Group-1 and was 100% (12/12) in Group-2.

Adrenal gland cortical hyperplasia was appreciated macroscopically in groups-1 and -2 in 23% (7/30) and 25% (3/12) of animals, respectively. Microscopically, this change was observed at a similar prevalence in 24% (5/21) of Group-1 animals and 25% (3/12) for Group-2. The high prevalence of cortical hyperplasia in both groups was a significant finding in the population. Grossly, congestion of the adrenal glands was observed in 23% (7/30) of Group-1 animals and 33% (4/12) of Group-2; however, histologic correlation was less frequent at 10% (2/21) and 17% (2/12), respectively. Adrenalitis and extramedullary hematopoiesis (EMH) were frequently observed in Group-2 animals involving the corticomedullary junction and depending on the degree may have resulted in a subgross change that could be interpreted as congestion grossly. Extramedullary hematopoiesis was observed microscopically in 10% (2/21) of Group-1 and 42% (5/12) of Group-2 animals. Adrenalitis, which was characterized by a mixed inflammatory cell infiltrate, was observed in 50% (6/12) of Group-2 animals; necrotizing adrenalitis was observed in 33% (4/12) of animals. Fibrosis and hemorrhage was observed in 25% (3/12) of animals. These changes were either not observed or occurred at a reduced frequency (hemorrhage 5%; 1/21) in Group-1 animals.

Macroscopically, thyroid gland changes consisted primarily of developmental or structural abnormalities including follicular and thyroglossal cysts which were observed more frequently in Group-2 at 8% (1/12) and 33% (4/12), respectively. In Group-1, 5% (1/19) of animals displayed both of these changes. On microscopic examination, follicular cysts were observed frequently in both groups with 36% (5/14) in Group-1 and 25% (3/12) in Group-2. Other common microscopic findings in Group-2 included congestion (68%; 8/12), colloid depletion/drop-out in 50% (6/12), hemosiderin deposition in 42% (5/12), and follicular papillary hyperplasia in 25% (3/12). Micronodular goiter was diagnosed in 17% (2/12) of animals when the combined characteristic changes of follicular hyperplasia, hemosiderin deposition, colloid depletion/drop-out and mineralization were observed. Thyroglossal duct cysts were found in 16% (5/31) of rough-toothed dolphins overall from this stranding.

Pituitary gland changes consisted primarily of developmental or structural abnormalities including follicular thyroidization in 100% of Group-2 animals (11/11) and 33% (1/3) of Group-1 animals, larger dilated follicular cysts (i.e., colloid cysts) in 27% (3/11) of Group-2 and 33% (1/3) in Group-1, and a Rathke's Pouch cyst (33% 1/3) in Group-1. Circulation disorder/disruption and intracellular material accumulation were also observed commonly in Group-2 animals each characterized by congestion in 36% (4/11) and adenohypophyseal mineralization in 27% (3/11) of animals, respectively. Generally, the aforementioned developmental changes would be considered incidental, non-specific findings; however, the high prevalence in both groups suggests a significant finding in this cohort, the relevance of which is unknown.

### Musculoskeletal System

The musculoskeletal system was grossly evaluated for both groups; however, microscopic findings were largely only available for Group-2. The prevalence for the gross changes from both groups is presented. In Group-2, musculoskeletal changes occurred at a higher prevalence in particular acute muscle necrosis (i.e., rhabdomyolysis) in 42% (5/12), subacute muscle necrosis (i.e., muscle pallor) in 33% (4/12) and vertebral scoliosis with muscle contracture in 25% (3/12). These changes represent a continuum of changes observed in the syndrome Capture/Exertional Rhabdomyolysis. In Group-1, rhabdomyolysis and vertebral scoliosis with muscle contracture were each observed in a single animal (1/34, 3%) while muscle pallor was not observed. The difference in prevalence between the two groups is consistent with the time course needed for the traumatic injury incurred during the stranding to become apparent clinically.

### Urogenital System

The total stranded population had a 74% (34/46) urinary system lesion prevalence which included primarily the kidney and urinary bladder and to a lesser extent ureters, urethra, and urine. Two animals from Group-1 had inconclusive findings (6%) while 10 animals were found to be without significant findings. On combined macroscopic and microscopic examination, in Group-1, the urinary system lesion prevalence was 69% (22/32) and was 100% (12/12) in Group- 2. The microscopic changes observed were generally very subtle and were characterized by inflammatory and degenerative changes primarily involving the glomeruli including Bowman's capsule thickening (hyalinization)/ membranous glomerular nephritis in 22% (6/27) from Group-1 and 42% (5/12) in Group-2, with membranoproliferative glomerulonephritis, glomerular synechia, glomerular obsolescence, and glomerular mineralization each in 8% (1/12). These changes are considered a declining continuum, the result of glomerular injury likely secondary to a chronic underlying immune mechanism. Other common findings which were only observed in Group-2 animals included medullary mineralization in 58% (7/12), tubular pigment deposition in 50% (6/12) and acute tubular necrosis (i.e., nephrosis) in 33% (4/12) of animals.

Reproductive gross lesions in Group-2 females consisted primarily of inflammatory and neoplastic changes. Focal, benign leiomyoma was observed in three animals from both groups combined. Incidentally, endometrial adenocarcinoma was observed in 22% (2/9) of Group-2 females. Grossly, two different patterns were observed including a raised, broad-based, focally-extensive endometrial mass and a diffuse tan circumferential thickening of both uterine horns and body. Neoplastic cells were similar in both neoplasms. In one case microscopically, the uterine horn endometrium was circumferentially thickened and effaced by an infiltrative mass characterized by cystic lobules with papillomatous projections lined by neoplastic polyhedral epithelial cells. Mitotic figures were uncommon, about one per high powered field. A non-encapsulated, well-circumscribed, mixed nodular, and multilocular neoplasm characterized the second case. The nodules consisted of pleomorphic polyhedral cells forming cords, acini and solid nests interspersed with fibrovascular stromal septa. The multiloculated cysts consisted of similar papillomatous projections lined by neoplastic simple columnar cells. Mitotic figures were common, one to two per high-powered field.

Clinically, hematuria and/or proteinuria are generally manifestations of glomerular abnormalities. In Group-2 animals, both these conditions were observed during post mortem urinalysis in 25% (3/12) of animals suggesting clinically significant glomerular disease as was observed in Group-2 animals (42%; 5/12 see above). Post mortem urinalysis was not conducted on Group-1 animals. In Group-2, the high prevalence of renal tubular epithelial cell pigment deposition (50%; 6/12) and acute tubular cell necrosis (33%; 4/12) could be attributed to the myoglobin and/or hemosiderin deposition causing tubular epithelial cell toxicosis and necrosis. Agonal hypoxia may have also contributed to the terminal acute tubular necrosis.

### Central Nervous System

The overall number of Group-1 and -2 animals with CNS lesions was 73% (30/41) central nervous system lesion which included findings in the cerebrum, cerebellum, midbrain, and spinal cord. Seven animals from Group-1 had inconclusive findings (23%; 7/30) and were further excluded while five animals were found to be without a significant finding by both gross and microscopic examination (i.e., normal). In Group-1, the central nervous system lesion prevalence was 83% (19/23) and in Group-2 it was 100% (11/11). Macroscopically, meningeal hemorrhages were found equally in both Group-1 and -2 animals (22 and 18%, respectively).

Microscopically, the majority of changes observed in both groups consisted of cerebral edema and inflammation. Cerebral edema is generally characterized by two pathogenic mechanisms, vasogenic edema and cytotoxic edema (9). There appears to be a division between the two groups such that findings associated with vasogenic edema were found more commonly, than those consistent with cytotoxic edema (i.e., anoxic injury), in Group-1. In Group-2, both types of changes were present although the vasogenic changes occur at a higher prevalence [e.g., perivascular edema and neuropil spongiosis, 75% (9/12)]. Myelin vacuolation (i.e., intramyelin edema) a characteristic changes suggestive of cytotoxic edema was observed in Group-1, 9% (2/23) and Group-2, 58% (7/12) of animals. Characteristic changes, observed commonly, consistent with vasogenic edema included neuropil spongiosis/vacuolation and perivascular edema both in 75% (9/12) of Group-2 animals whereas for Group-1 neuropil spongiosis/vacuolation was in 26% (6/23) and perivascular edema was in 39% (9/23).

Additionally, perivascular hemorrhage, intracerebral vascular congestion and choroid plexus edema were noted commonly in Group-2 animals in 25% (3/12) while in Group-1 perivascular hemorrhage was seen in 22% (5/23). Hemorrhage involving the meningeal vasculature occurred in 75% (9/12) of Group-2 animals and less commonly in Group-1 (13%, 3/23). Meningeal edema was observed commonly in Group-2 animals at 42% (5/12) and occurred in 9% of Group-1 animals. Hemosiderin laden macrophages (i.e., siderophages) associated with resolving hemorrhage were present more commonly in Group-2 animals at 25% (3/12) and less commonly in Group-1 animals (13%, 3/23). Incidentally, there were commonly observed golden to black spicules within glial cell cytoplasm for Group-2 at 25% (3/12) which may be hematin; however, special histochemical stains may be beneficial in further characterizing these pigments. Meningeal fibrosis which may be either a reparative or age related change was observed commonly in 58% (7/12) of animals in Group-2 and in only one from Group-1 (4%).

Inflammatory changes in the central nervous system were commonly observed in Group-2 animals which were characterized by gliosis (58%, 7/12), non-suppurative meningitis (42%, 5/12) that consisted of mostly lymphocytes and/or plasma cells and microglial nodules (25%, 3/12). Demyelination in Group-1 was observed commonly in both the cochlear nucleus and eighth cranial nerve (CN-8) (30%, 7/23), while collectively in Group-2 there were various degenerative changes in CN-8 including myelin vacuolation, myelin hyalinization, axonal spheroids, and edema (5/8, 63%). Other clinically significant inflammatory changes observed less commonly included a case of fungal meningoencephalitis (morphologically consistent with *Penicillium* sp. which was isolated from the animal's pharynx), and two cases of trematodiasis one from each group. Only one case in Group-1 presented with clinically significant CNS lesions for which culture results were available. The case presented with macroscopic cavitary meningoencephalitis that was characterized microscopically as a non-suppurative, hemorrhagic meningoencephalitis with cholesterol granulomas and dystrophic mineralization from which a mixed growth of *Enterobacter cloacae, E. coli, Cronobacter* (formerly *Enterobacter*) *sakazakii, Micrococcus* spp. and an unidentifiable coryneform gram positive bacillus were isolated.

## Discussion

In the investigation of this mass stranding event, incidental and significant lesions were found in the two groups. Given that this event involved multiple prosectors and histopathologists, discrepancies between lesion prevalence for gross and microscopic changes in the same organs did occur, but had limited impact on the overall evaluation. Overall, Group 1 animals had congestion and edema related to terminal cardiopulmonary collapse (shock) and euthanasia and Group 2 animals had secondary infections likely related to stress-related diminished immune function.

The overall body condition of the animals initially declined for Group-2 with a reduced blubber layer and axial musculature. However, findings from Group-1 suggest animals were anorectic and in a subclinical energy deficient state including pancreatic zymogen granule depletion, hypodermal adipose tissue atrophy and thin body condition. In the released rehabilitated animals, Karns et al. (10) found a statistically significant difference between intake and release body mass index for individual from this stranding indicating a positive weight gain for all but one surviving animal.

### Common Group Findings

Common findings to the group involved the endocrine, hemolymphatic, female reproductive, hepatobiliary systems and central nervous system. Specifically, there were thyroglossal duct cysts, uterine leiomyomas, thyroglossal cysts, thymic involution, adrenal cortical hyperplasia, cranial nerve VIII degradation, and biliary cystadenomas. These findings could indicate background lesions in not only the stranded population, but also the species. Some of these lesions occurred in >20% of animals either when the groups were evaluated separately or combined for findings that were not time dependent or impacted by medical treatment. The thyroglossal cyst is a benign developmental change has been described in 15% (9/60) of individually stranded bottlenose dolphins along the Texas Coast from 1991 to 2005 (11); the rough-toothed dolphins had a similar prevalence.

Uterine leiomyomas have been observed in various cetacean species (12) and while benign, may, depending on size and location, affect reproductive productivity/success. Biliary cystadenomas is uncommon benign biliary tumor that has not previously been observed in stranded *Tursiops* (13) or other odontocetes (14) and could result from an exuberant fibroadenomatous inflammatory response associated with bile duct trematodiasis. Thymic involution with or without thymic cysts occurs in aged animals. Physiologic thymic involution and cyst development have been described in other odontocetes (e.g., harbor porpoises, bottlenose dolphins) (15, 16). The high rate of occurrence in this pod of animals could be attributed to the high number of adult animals in the stranding cohort (10). While thymic involution was considered to be an age-related change in this pod, pre-mature thymic and lymph node lymphoid depletion can occur with malnutrition, stress induced elevated cortisol, or immune suppression due to infection.

Thyroid follicular cysts suggests a hyperplastic response due to a nutritional deficiency. Thyroid gland follicle colloid depletion has also been described in sick stranded (11) and captive bottlenose dolphins (17) and was described in association with perinatal hyperplastic goiter in captive born bottlenose dolphins (18). The relevance of this finding in this pod is currently unknown. Lee et al. (19) described degenerative thyroid gland changes (i.e., follicular epithelium degeneration with mild attenuation and reduced colloid) and elevated thyroid stimulating hormone induced hyperthyroidism in rats with exposure to high doses of the flame retardant polybrominated diphenyl ethers. A survey of the thyroid gland from stranded bottlenose dolphins along the Texas Coast found that colloid filled follicular cysts in 7–8% of animals, while squamous lined cysts (suggestive of a thyroglossal duct cyst) were seen in 15% of animals from 1991 to 2005 (11) which was similar to the prevalence found in this pod (16%).

The adrenal gland changes consisted of chronic defects of growth (i.e., cortical hyperplasia, and EMH), acute inflammation or chronic repair, and acute vascular defects. Adrenal cortical hyperplasia has been attributed to chronic stress in bottlenose dolphins (20) and disease in harbor porpoises (21). Lair et al. (22) suspected stress in causing cortical enlargement in stranded beluga.

CN-8 degenerative changes, some with involvement of the Cochlear Nucleus, which, may be related to an anterograde inflammatory process, associated with either a cranial sinus or middle-inner ear infection that ascended the nerve as in verminous sinusitis (e.g., *Stenurus* sp. or *Nasitrema* sp.). Parasitogenic eighth cranial neuropathy characterized by CN-8 degeneration due to perivascular edema has been described in other odontocetes; however, changes in the Cochlear Nucleus have not previously been described associated with this syndrome (23, 24). Retrograde cochlear nerve degeneration has been documented in cats and humans associated with hypoxia and acoustic trauma (i.e., overstimulation); however, to the authors' knowledge this change has not been documented in delphinids (25, 26). In (27) clinical audiogram studies on various stranded dolphins documented severe hearing loss in other delphinids including rough-toothed dolphins, although hearing loss was not found in the few individuals examined from this stranding event.

### Infectious Disease

Bacterial and fungal infections were observed in Group-1 and -2. Viral infection with molecular sequencing was limited to the oropharyngeal lesions. Mixed bacterial isolates were isolated in some cases, which may reflect secondary infection or overgrowth. In Group-1, there was a bacterial meningoencephalitis and mixed fungal and bacterial pneumonia. For the meningoencephalitis, primary trematode migration (e.g., *Nasitrema* sp.) or fungal encephalitis (28) were suspected. Due to the field conditions, cultures were not conducted.

In Group-2, a *Penicillium brevicompactum*, isolated from the pharynx caused a leptomeningitis and bronchopneumonia. *Morganella morganii* and *Staph. aureus* were also isolated from the pharynx. *Penicillium* species are an ubiquitous fungi not previously documented in dolphins, and not considered a virulent, invasive pathogen like *Aspergillus* species (29), however, in a compromised animal, infection would be possible. *Penicillium brevicompactum* infection has been documented in an immunosuppressed allogeneic bone marrow transplant recipient with an invasive lung infection (30). The isolation of *Staph. aureus* even in mixed culture from the respiratory system in a Group-2 animal would be of concern as it is considered a high risk pathogen in dolphins in managed care (31, 32).

*Mycobacterium mucogenicum* was isolated from the prescapular lymph node and ureter from one dolphin in Group-2 suggesting systemic infection. *Mycobacterium mucogenicum* is ubiquitous in the environment and generally associated with nosocomial infection in humans, both immunocompetent and immunosuppressed (33) and has not previously been reported in a dolphin.

Bronchopneumonia was observed in 6 dolphins from Group-1 suggesting that some animals were compromised prior to the stranding event. In Group-2, the increased occurrence of bronchopneumonia associated with infectious causes not present in the population at the time of stranding suggests that while some may have entered rehabilitation with pneumonia it is probable that some animals developed pneumonia while in rehabilitation or possibly secondary to stranding-related aspiration. Howard et al. (34) asserted that the manifestation of bacterial infections in free-ranging dolphins could also be stress associated once in captivity or be intercurrent infections to other disease processes.

The pure isolate of *Citrobacter freundii*, a facultative anaerobic gram-negative bacteria of the family *Enterobacteriaceae* (35), which has not previously reported in dolphins from a Group-2 case of bronchopneumonia is interesting as it is an uncommon opportunistic pathogen which has been isolated, from a marine turtle in rehabilitation (36). Additionally, organisms obtained on culture included predominantly members of the family *Enterobacteriaceae*, fecal streptococci (i.e., *Enterococcus* spp.) and marine commensals (i.e., *Pseudomonas* sp. and *Vibrio* sp.) which could suggest that the exposure of these animals to environmental contaminants/ pathogens was a major contributor to their respiratory infections (36–38). Respiratory infections associated *Vibrio damselae* have been documented in stranded dolphins and from skin wounds in animals held in open ocean pens (39–41).

Pharyngeal plaques were observed grossly in a total of 6 of 9 animals from both groups. Gammaherpesvirus was found in lesions located in the oropharyngeal using conventional and real-time PCR assays that target the DNA polymerase and the glycoprotein B gene sequences of members of the *Herpesviridae* family of viruses. The strong positive signals for a gammaherpesvirus obtained with DNA extracted from pharyngeal and epiglottal plaques suggests active infection. While the presence of the virus solely in association with similar lesions across multiple individuals of the same pod supports the likelihood that the identified gammaherpesvirus was causal for these mucosal plaques and is consistent with horizontal transmission. Similar gammaherpesviruses have been identified by molecular analysis with sequencing data suggesting cetacean specific gammaherpesvirus infections in hyperplastic mucosal genital and oral lesions, and skin of stranded cetaceans (4, 42, 43). Whether these viral infections suggest impacted population health is speculative as other viruses, such as poxvirus, are fairly ubiquitous in cetaceans.

### Neoplasia

In Group-2, malignant neoplasia was observed but was focal and not related to the cause of death in the cases it was found in. Incidentally, a subclinical nasopharyngeal scirrhous ductal carcinoma, which has not previously been reported in small odontocetes, was found in the parapharyngeal skeletal musculature. Two forms of non-metastatic, malignant uterine adenocarcinoma were observed. One consisted of diffuse multiloculated cysts lined by fibropapillary projections and the other a focal, mixed nodular and multiloculated mass. Uterine adenocarcinoma has been documented in a single stranded bottlenose dolphin (44) and beluga whale (*Delphinapterus leucas*) (45).

### Non-inflammatory Lesions

Lesions in the respiratory system consistent with vascular defect including microscopic pulmonary edema and gross pulmonary congestion/hemorrhage were appreciated at a higher prevalence in Group-1 animals which may be attributed to the nature of their deaths either due to euthanasia or terminal cardiovascular collapse (shock).

In Group-2, there was an elevated number of cases with features characteristic of hemorrhagic diathesis including hemosiderosis (67%) and hemorrhage drainage (83%) which may have resulted in increased hematopoiesis and subsequent increased activity in the spleen and recruitment of sites of extramedullary hematopoiesis in the adrenal gland, liver, and lung. This regenerative response across Group-2 was clinically significant finding suggesting that the group had myelostimulatory disease processes present such as hemorrhage, red blood cells consumption/destruction—resulting in concurrent anemic hypoxia or increased tissue inflammation and repair (46). Adrenal gland extramedullary hematopoiesis is occasionally observed in the cortex of other mammalian species (46). Interestingly, adrenal gland EMH was not been observed in singly stranded *Tursiops* from the Florida coast in a retrospective survey of animals of varying age classes, 1996–2004 (13). Adrenalitis was commonly observed, however, a common etiology could not be determined by routine histologic examination (e.g., viral, protozoa, bacteria, or fungal).

In the CNS, there was a high prevalence of cerebral edema across groups which could be related to inflammatory/infectious disease resulting in vascular damage and endothelial cell leakage possibly related to bacteremia, toxemia, or shock. Vasogenic edema is primarily the result of cerebral blood vessel damage and subsequent leakage as in infection. Cytotoxic edema results from glial cell injury and osmoregulation functional disruption as in hypoxia and ischemia etc. (47). According to Girolami et al. (9) in generalized edema elements of both types are present. Specifically in Group-1, CNS changes consistent with vasogenic edema occurred commonly but were found more frequently in Group-2. In Group-2, CNS changes consistent with both cytotoxic and vasogenic mechanisms of edema occurred more frequently (e.g., neuronal satellitosis and anoxic changes, verses neuropil vacuolation and perivascular edema, respectively) which could reflect the varying systemic or agonal changes terminally between the two groups.

Acute necrotizing pancreatitis was a significant clinical finding that can be a life-threatening disease. The concern during this event was that the condition was found at a high prevalence, observed in both groups from the time of stranding into the early phases of rehabilitation. Commonly, pancreatic acinar cell zymogen granule depletion (i.e., acinar atrophy) was observed from both groups and was therefore considered a significant finding in this population. The high prevalence in Group-1 animals suggests that the condition was common in the pod at the time of stranding. Interestingly, the change was observed at a higher rate in Group-2 animals (i.e., 25% Group-1 vs. 73% Group-2). The cause of the pancreatitis was investigated to determine if the disease was communicable and the result of a common contagion or infestation.

The etiology for acute necrohemorrhagic pancreatitis in dolphins is unknown. Likewise, the pathogenesis of acute pancreatitis is poorly understood in humans, other domestic species and laboratory animals. However, there are pre-established potential causes in humans and other species that can be divided into three main categories including: duct obstruction (e.g., parasitism, inflammation, and lithiasis), acinar cell injury (e.g., drugs, ischemia, viruses, oxidative), and defective intracellular transport (e.g., metabolic injury/disorder) (48).

The obstruction of the pancreatic ducts or the shared convergent bile duct due to an ascending duct infection/inflammation from trematodiasis, cholangitis, or duodenitis was considered due to the presence of *Campula* sp. and *Pholeter* sp. in some individuals from this event. However, the likelihood that all animals with pancreatitis experienced parasite induced duct obstruction and reflux was small. Although inflammation and obstruction maybe contributory in certain cases, additional analysis would have to be applied to better determine the level of association between these findings and the disease process.

In humans, a high percentage of acute pancreatitis cases are considered idiopathic. The high prevalence in this population of stranded dolphins suggests a shared cause and/or common pathogenesis. Factors, which may be causal, that were also found in high prevalence during this investigation include parasitism and anorexia (i.e., zymogen granule depletion) or potentially a combination of these factors. Thomas et al. (49) found no conclusive evidence of a direct causative role for diet in the development of acute pancreatitis in humans; however, there was an indication that diet maybe a cofactor and the reintroduction of feeding after prolonged anorexia especially in malnourished individuals may lead to metabolic disorders and/or nutritional/malnutritional pancreatitis. Low-protein high fat diets, especially in dogs, resulting in hyperlipidemia has been shown to be associated with pancreatitis possibly due to the generation of toxic fatty acids by the activation of lipase (50).

Ultimately, it was theorized that the condition was due to a non-infectious cause and was the culmination of either a metabolic abnormality (i.e., reduced secretion of pancreatic enzymes as a result of zymogen granule depletion) or growth defect (i.e., acinar atrophy) which was possibly caused by prolonged inanition of the entire pod. Generally, acinar cell zymogen granule depletion is associated with prolonged inanition/starvation (50). This preexisting condition was then likely exacerbated by the re-introduction of high caloric lipid-rich fish during rehabilitation resulting in life-threatening acute necrotizing pancreatitis. As a result, animal husbandry changes were made during rehabilitation to reconfigure the dietary feeding regimen to food items with less fat and higher protein content which halted mortalities attributed to necrotizing pancreatitis. The period of anorexia prior to the development of pancreatic zymogen granule depletion is unknown in any species of dolphin. Therefore, the period of time the pod spent “out-of-habitat” and away from normal prey items cannot be ascertained. In the end, a high prevalence of non-specific changes including decreased body condition, hepatic lipidosis/vacuolar hepatopathy, and pancreatic zymogen granule depletion suggest decreased food intake and a negative energy balance.

Additional studies are needed to further determine the level of significance based on confounding host variables (e.g., age, length class, or gender) as well as multivariate analysis to determine the strength of association between putative factors and the observed lesion/disease. The effort to split the stranded population into two groups based on rehabilitative effort was done to remove or control for rehabilitation as a confounding variable. This division was also done to determine if there was a difference in the mortality between the two groups. In Group-1, individually there were many cases with various levels of morbidity involving several organ systems that either alone or in concert were not of the expected severity to result in marked debilitation let alone spontaneous death. Other causes of morbidity and mortality to consider which would not be apparent by post-mortem examination would include metabolic derangements for example metabolic acidosis secondary to exertional rhabdomyolysis, electrolyte imbalance secondary to dehydration and anorexia. Lesions observed in Group-2, which may have contributed to animal mortality in this group include moderate to severe acute necrotizing pancreatitis, followed by various bacterial infections associated with environmental contamination, and renal nephrotoxicosis secondary to myoglobin or hemoglobin nephropathy associated with exertional rhabdomyolysis. Ultimately, the entire pod suffered from the detrimental effects associated with the mass stranding event and prolonged inanition. Findings from this large cohort provide a better understanding of the natural history and diseases in rough-toothed dolphins.

## Data Availability Statement

The datasets generated for this study can be found in the GenBank NCBI accession number: KX424962; https://www.ncbi.nlm.nih.gov/nuccore/1109522396 and GenBank NCBI accession number: MT03845; https://www.ncbi.nlm.nih.gov/nuccore/MT038045.1.

## Ethics Statement

Ethical review and approval was not required for this animal study because study material was collected from salvaged stranded rough-toothed dolphins that were humanely euthanized or died spontaneously after stranding. These samples were collected and analyzed under USDOC, NMFS Scientific Research Permit 932-1489-08.

## Author Contributions

All contributing authors have approved this work for publication. RE, DR, WM, AC, GL, AS, and GB: sample data acquisition. RE, DR, and GB: sample histopathologic analysis and interpretation and original manuscript preparation. CR: PCR and sequencing analysis. WM, AC, GL, AS, and CR: manuscript review. The final revised manuscript was posthumously published after the death of co-author, friend, and colleague GB.

## Conflict of Interest

The authors declare that the research was conducted in the absence of any commercial or financial relationships that could be construed as a potential conflict of interest.

## Supplementary Material

Supplementary Figure 1 Lung acute diffuse severe edematous necrosuppurative bronchopneumonia, rough-toothed dolphin (case Y375). Alveoli filled with mixed inflammatory cell debris and pink proteinaceous material (edema) with moderate numbers of intralesional rod bacteria (HE). Obj. 4x. Bar = 200 μm.

Supplementary Figure 2 Oropharyngeal mucosal plaque, rough-toothed dolphin (case R356). Oropharynx has a focal slightly raised corrugated firm tan plaque.

Supplementary Figure 3 Pancreas, necrohemorrhagic pancreatitis, rough-toothed dolphin (case Y375). The pancreas has marked loss of exocrine pancreatic structures with dissecting interlobular and perivascular extravasated erythrocytes (HE). Obj. 4x. Bar = 200 μm.
