# Supplementary figures and images for: Macroscopic and Histopathologic Findings From a Mass Stranding of Rough-Toothed Dolphins (Steno bredanensis) in 2005 on Marathon Key, Florida, USA
Source: Front Vet Sci. 2020 Sep 2;7:572. doi: 10.3389/fvets.2020.00572 (PMC7492606; doi:10.3389/fvets.2020.00572)

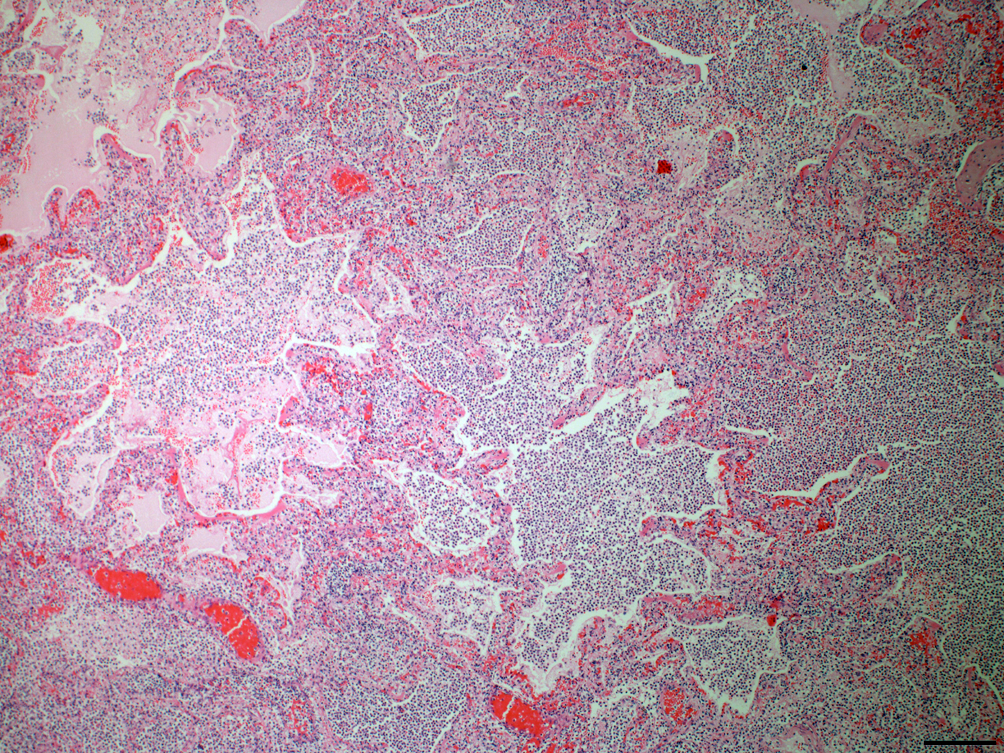

Supplement: Supplementary Figure 1 — Lung acute diffuse severe edematous necrosuppurative bronchopneumonia, rough-toothed dolphin (case Y375). Alveoli filled with mixed inflammatory cell debris and pink proteinaceous material (edema) with moderate numbers of intralesional rod bacteria (HE). Obj. 4x. Bar = 200 μm. [file Image_1.TIF]

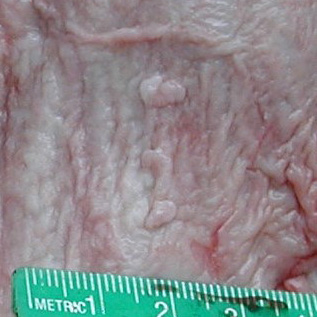

Supplement: Supplementary Figure 2 — Oropharyngeal mucosal plaque, rough-toothed dolphin (case R356). Oropharynx has a focal slightly raised corrugated firm tan plaque. [file Image_2.JPEG]

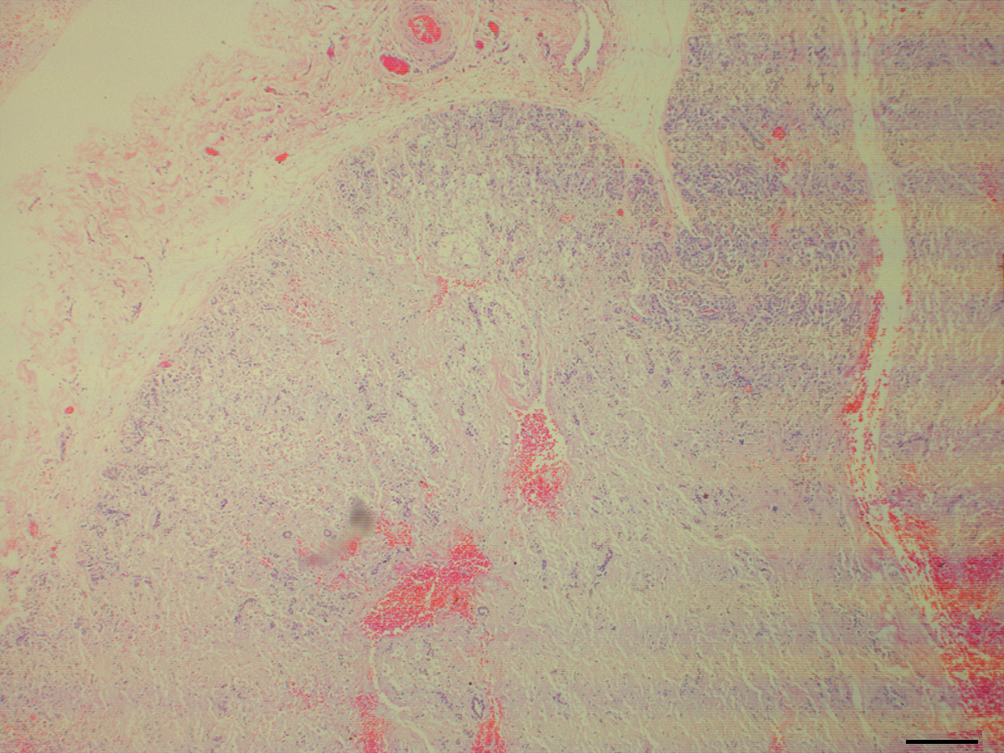

Supplement: Supplementary Figure 3 — Pancreas, necrohemorrhagic pancreatitis, rough-toothed dolphin (case Y375). The pancreas has marked loss of exocrine pancreatic structures with dissecting interlobular and perivascular extravasated erythrocytes (HE). Obj. 4x. Bar = 200 μm. [file Image_3.TIF]
